# Multifactorial Shock: A Neglected Situation in Polytrauma Patients

**DOI:** 10.3390/jcm11226829

**Published:** 2022-11-18

**Authors:** Jialiu Luo, Deng Chen, Liangsheng Tang, Hai Deng, Cong Zhang, Shunyao Chen, Teding Chang, Liming Dong, Wenguo Wang, Huaqiang Xu, Miaobo He, Dongli Wan, Gang Yin, Mengfan Wu, Fengsheng Cao, Yang Liu, Zhao-Hui Tang

**Affiliations:** 1 Department of Trauma Surgery, Tongji Trauma Center, Tongji Hospital, Tongji Medical College, Huazhong University of Science and Technology, Wuhan 430030, China; 2 Intensive Care Unit, Trauma Center, Suizhou Central Hospital, Hubei University of Medicine, Suizhou 441300, China; 3 Department of Trauma Surgery, Trauma Center, Tianmen First People’s Hospital, Hubei University of Science and Technology, Tianmen 417300, China; 4 Department of Emergency and Intensive Care, Trauma Center, Xiangyang Central Hospital, Affiliated Hospital of Hubei University of Arts and Science, Xiangyang 441021, China

**Keywords:** polytrauma, shock, multifactorial shock, retrospective study

## Abstract

Background: Shock after traumatic injury is likely to be hypovolemic, but different types of shock (distributive shock, obstructive shock, or cardiogenic shock) can occur in combination, known as multifactorial shock. Multifactorial shock is a neglected area of study, and is only reported sporadically. Little is known about the incidence, characteristics, and outcomes of multifactorial shock after polytrauma. Methods: A retrospective, observational, multicenter study was conducted in four Level I trauma centers involving 1051 polytrauma patients from June 2020 to April 2022. Results: The mean Injury Severity Score (ISS) was 31.1, indicating a severely injured population. The most common type of shock in the early phase after polytrauma (≤48 h) is hypovolemic shock (83.2%), followed by distributive shock (14.4%), obstructive shock (8.7%), and cardiogenic shock (3.8%). In the middle phase after polytrauma (>48 h or ≤14 days), the most common type of shock is distributive shock (70.7%), followed by hypovolemic shock (27.2%), obstructive shock (9.9%), and cardiogenic shock (7.2%). Multifactorial shock accounted for 9.7% of the entire shock population in the early phase and 15.2% in the middle phase. In total, seven combinations of multifactorial shock were described. Patients with multifactorial shock have a significantly higher complication rate and mortality than those with single-factor shock. Conclusions: This study characterizes the incidence of various types of shock in different phases after polytrauma and emphasizes that different types of shock can occur simultaneously or sequentially in polytrauma patients. Multifactorial shock has a relatively high incidence and mortality in polytrauma patients, and trauma specialists should be alert to the possibility of their occurrence.

## 1. Introduction

Trauma is a leading cause of global mortality and accounts for 10.1% of the global burden of disease. Annually, nearly 4.8 million people die from injuries [1,2,3]. Polytrauma accounts for only 3–5% of total trauma, which is characterized by poorer outcomes and a higher mortality rate, primarily because of fatal damage and intractable complications, resulting in prolonged hospitalization and recovery periods with immense social and economic implications [4,5]. Shock is the most common complication in polytrauma, which is reportedly the first leading cause of death in these patients [6,7,8]. According to the European Society of Intensive Care Medicine (ESICM) guidelines, identifying the type of shock is paramount to improving the target causal and supportive therapies [9]. Depending on the etiology and characteristics of hemodynamics, shock patients can be roughly divided into the following four shock subtypes: distributive, hypovolemic, cardiogenic, and obstructive. Specific therapeutic measures are needed for the different types of shock, owing to differences in their pathogenesis and pathophysiology [9,10,11]. For example, obstructive shock needs immediate causal treatment; for example, pericardial tamponade in polytrauma patients can be relieved quickly by pericardiocentesis drainage. Other treatments for shock (e.g., fluid resuscitation) are ineffective for this condition. Therefore, the prompt identification of the types of shock in polytrauma patients is essential to initiate aggressive management.

Shock after severe traumatic injury is likely to be hypovolemic, but different types of shock can occur in combination [12]. Multifactorial shock is diagnosed when multiple shock types overlap [13,14,15,16]. Multifactorial shock is a neglected area of study in polytrauma, and only reported as sporadically [17,18]. Little is known about the incidence, characteristics, and outcomes of multifactorial shock after polytrauma. 

Therefore, we carried out this observational, retrospective, and multicenter study, involving 1051 polytrauma patients. The primary outcome was the incidence of various types of shock in different phases after polytrauma, especially multifactorial shock; the secondary end points were to describe the clinical outcome parameters of multifactorial shock in polytrauma patients.

## 2. Materials and Methods

### 2.1. Patient Selection

A retrospective, observational, multi-center trial was performed using the medical records for all trauma patients admitted to four Level I trauma centers (verified by China Trauma Rescue & Treatment Association (CTRTA)) from June 2020 to April 2022. The inclusion criteria were as follows: (1) age > 18 years and (2) should have met the new “Berlin definition” of polytrauma: two injuries that are ≥3 on the abbreviated injury score (AIS) and one or more additional diagnoses (pathologic condition), that is, hypotension (systolic blood pressure ≤ 90 mmHg), unconsciousness (Glasgow Coma Scale [GCS] score ≤ 8), acidosis (base deficit ≤−6.0), coagulopathy (partial thromboplastin time [PTT] ≤ 40 s or international normalized ratio [INR] ≥ 1.4), and age ≥ 70 years) [19]. The exclusion criteria were: (1) an admission time of more than 6 h after trauma; (2) previous medical history of malignancy or metabolic, consumptive, and immunological diseases; and (3) missing clinical records. During the study period, a total of 1379 polytrauma patients were admitted to the trauma centers, of whom 1051 consecutive patients met the eligibility requirements. 

### 2.2. Diagnostic Modalities

Shock was diagnosed according to the diagnostic guidelines of the ESICM [9]. The total observation period was 28 days. To investigate the incidence of shock in different phases after polytrauma, we defined two observation phases. The early phase was defined as the period of ≤48 h after polytrauma and the middle phase was defined as the period between 48 h and 14 days [6]. According to the characteristics of hemodynamics, shock can be divided into the following four types: hypovolemic shock, distributive shock, cardiogenic shock, and obstructive shock [20]. Hypovolemic shock (HS) is defined as a condition of inadequate organ perfusion caused by loss of intravascular volume and diagnosed in accordance with the guidelines [21,22]. Distributive shock (DS) is defined as a stage of relative hypovolemia resulting from the pathological redistribution of the absolute intravascular volume and can be divided into three subtypes: septic shock, neurogenic shock, and anaphylactic shock [10]. Septic shock is diagnosed by a vasopressor requirement to maintain mean BP 65 mm Hg or greater and serum lactate level greater than 2 mmol/L (>18 mg/dL) after adequate fluid resuscitation [23,24,25]. Neurogenic shock is defined as a state of imbalance between the sympathetic and parasympathetic regulation of cardiac action and vascular smooth muscle and diagnosed according to the latest diagnostic guidelines [26,27,28]. Anaphylactic shock is defined as a severe, life-threatening, and systemic hypersensitivity reaction and diagnosed according to the diagnostic guidelines [29,30]. Cardiogenic shock (CS) is defined as a state in which ineffective cardiac output caused by a primary cardiac disorder results in both clinical and biochemical manifestations of inadequate tissue perfusion and is diagnosed according to the American Heart Association guidelines [31,32,33,34]. Obstructive shock (OS) is defined as a condition caused by the obstruction of the great vessels or the heart itself and diagnosed according to relevant international diagnostic guidelines [10,35,36]. Multifactorial shock is defined as experiencing more than one type of shock and was diagnosed on the basis of the studies cited [13,14,15,16]. Undifferentiated shock is diagnosed as a situation where shock is recognized but these shock patents cannot be classified into any of the mentioned types of shock [37,38]. The severity of shock was diagnosed according to the modified shock index (MSI; the ratio of heart rate to mean arterial pressure). Whenever present, the severity of shock was recorded as mild shock (1.0 < MSI < 1.5), moderate shock (1.5 ≤ MSI < 2.0), or severe shock (MSI ≥ 2.0) [39,40].

A final clinical shock diagnosis was confirmed through the agreement of three independent specialists in trauma surgery or critical care medicine. In a plenary meeting, the committee of auditors discussed the cases where the three independent experts were unable to reach a consensus. When the plenary discussion did not reach full consensus, the matter was resolved by the majority. All enrolled subjects received standardized treatment and management per established shock and polytrauma guidelines [9,41].

### 2.3. Data Collected

Patient data collected included the following: age; sex; mechanisms of injury; injury regions; Glasgow coma scale (GCS); Injury Severity Score (ISS); laboratory values; imaging examination; and discharge record. Events of the hospital course were recorded such as complications including acute respiratory distress syndrome (ARDS), venous thrombotic events (VTE), acute gastrointestinal injury (AGI), acute kidney injury (AKI), hospital acquired infections (HAI), multiple organ failure (MOF), length of ICU stay, duration of ventilator use, use of vasoactive agents, and death. 

### 2.4. Study End Points

The primary outcome was the incidence of various types of shock in different phases after polytrauma, especially multifactorial shock; the secondary end points were to describe the clinical outcome parameters of multifactorial shock in polytrauma patients.

### 2.5. Ethical Statement

This study was approved by the Tongji Hospital Institutional Review Board at the Huazhong University of Science and Technology (IRB number: TJ-IRB20200720; approval date: 22 July 2020). Each study site obtained approval from their local review board: Tianmen First People’s Hospital Institutional Review Board (IRB number: 2020010097; approval date: 8 January 2020), Suizhou Central Hospital Institutional Review Board (IRB number: none; approval date: 1 June 2019), and Xiangyang Central Hospital Institutional Review Board (IRB number: none; approval date: 10 October 2021). The study was conducted according to the guidelines of the Declaration of Helsinki. Informed consent was obtained from each patient or the patient’s legally authorized representative involved in the study.

### 2.6. Statistical Analysis

Data were analyzed with SPSS 23.0 (SPSS Inc., Chicago, IL, USA) and presented by GraphPad Prism software 9.3.1 (GraphPad Software Inc., San Diego, CA, USA) and Origin software 2021 (Origin Lab Inc., Northampton, MA, USA). Prior to analysis, all data were examined for normality and homogeneity of variance. Continuous variables are presented as means ± standard deviation (SD). Categorical variables are presented as frequency counts and percentages. Differences between groups on continuous data were analyzed using one-way analysis of variance (ANOVA) followed by Bonferroni test or Kruskal–Wallis test for non-parametric data. Group differences in categorical variables were compared with using Pearson’s chi-square or Fisher’s exact test for non-parametric data. *p* < 0.05 indicates statistically significant difference.

## 3. Results

### 3.1. Characteristics of Polytrauma Patients

**Figure 1:**
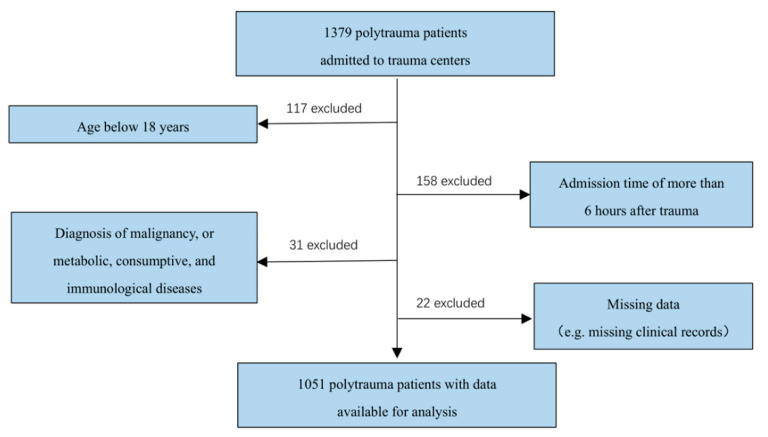
Case identification procedure.

From June 2020 to April 2022, a total of 1379 polytrauma patients were admitted to the trauma centers, of whom 1051 consecutive patients met the eligibility requirements (Figure 1). The demographics and characteristics of these patients are shown in Table 1. The mean age of the cohort was 50.2 ± 13.7 years, and patients were predominantly male (75.4%). The mean Injury Severity Score (ISS) was 31.1 (range 18 to 48), indicating a severely injured population. Overall, 69.1% (726/1051) patients suffered head injury, 62.6% (658/1051) patients had a limb injury, 55.1% (579/1051) suffered spine injury, and 46.4% (488/1051) suffered chest injury, while abdominal injury and pelvic injury were recorded for 33.7% (354/1051) and 29.6% (311/1051) patients, respectively. The injury was mainly caused by traffic accident (48.7%; 512/1051), followed by falls (44.8%; 471/1051), crush (4.6%; 48/1051), and other types of accidents (1.9%; 20/1051).

### 3.2. Incidence of Shock in Polytrauma Patients

The overall incidence of shock among all enrolled trauma patients in the early phase (≤48 h) after polytrauma was 74.7% (785/1051). Seventy-two patients died in the early phase after polytrauma. Of the surviving 979 patients, 34.2% (335/979) patients were diagnosed with shock in the middle phase (>48 h or ≤14 days) after polytrauma. The incidence of shock was significantly different (74.7% vs. 34.2%; *p* < 0.01) between the two phases.

The severity of shock was graded based on MSI. In the early phase, mild shock accounted for 19.0% (149/785), moderate shock for 41.9% (329/785), and severe shock for 39.1% (307/785). In the middle phase, mild shock accounted for 13.4% (45/335), moderate shock for 39.7% (133/335), and severe shock for 46.9% (157/335). The incidence of severe shock was significantly higher in the middle phase than in the early phase (46.9% vs. 39.1%; *p* < 0.05).

All enrolled patients were divided into two groups: the shock group and the non-shock group. The baseline characteristics of the two groups are shown in Table 2, wherein no significant difference was observed between the two groups with respect to age, sex, mechanisms of injury, injury region, and GCS scores (*p* > 0.05). The overall mortality was 6.9% (72/1051) in the early phase after polytrauma; 68 patients died in the shock group (8.7%; 68/785), and four patients died in the non-shock group (1.5%; 4/266). The overall mortality was 2.6% (25/979) in the middle phase after polytrauma; 20 patients died in the shock group (6.0%; 20/335), and five patients died in the non-shock group (0.8%; 5/644). The majority of deaths occurred in the shock group during the early phase (8.7% vs. 1.5%; *p* < 0.01) and middle phase (6.0% vs. 0.8%; *p* < 0.01) after polytrauma.

### 3.3. Incidence of Various Types of Shock in Polytrauma Patients

In the early phase after polytrauma, the most common type of shock was hypovolemic shock (83.2%; 653/785), followed by distributive shock (14.4%; 113/785), obstructive shock (8.7%; 68/785), and cardiogenic shock (3.8%; 30/785). During this period, all patients with distributive shock were diagnosed with neurogenic shock. 

The most frequently encountered type of shock in the middle phase after polytrauma was distributive shock (70.7%; 237/335), followed by hypovolemic shock (27.2%; 91/335), obstructive shock (9.9%; 33/335), and cardiogenic shock (7.2%; 24/335). Among the patients diagnosed with distributive shock, 65.4% (219/335) had septic shock, 19.4% (65/335) had neurogenic shock, and 0.3% (1/335) had anaphylactic shock (Table 3).

**Figure 2:**
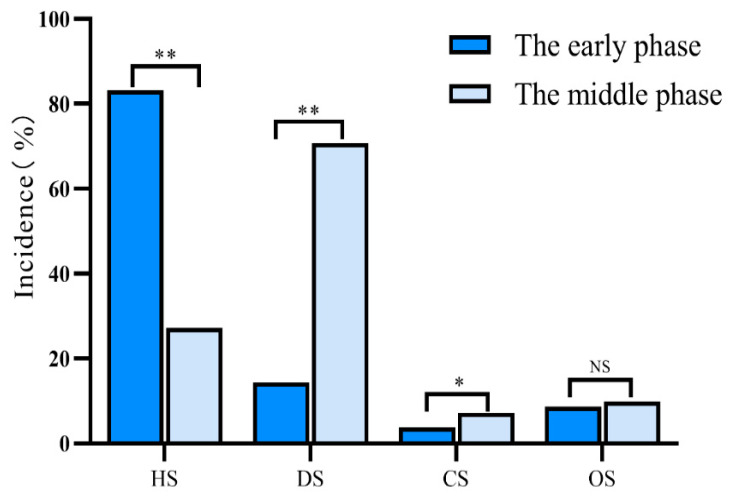
Incidence of different types of shock in polytrauma patients. The early phase was defined as the period of ≤48 h after polytrauma and the middle phase was defined as the period between 48 h and 14 days. HS: hypovolemic shock; DS: distributive shock; CS: cardiogenic shock; OS: obstructive shock; * *p* < 0.05; ** *p* < 0.01; NS: not statistically significant.

In our study, we compared the incidence of each type of shock at different time periods to analyze its changes over time. There were significant differences in the incidence of hypovolemic shock (83.2% vs. 27.2%; *p* < 0.01), distributive shock (14.4% vs.70.7%; *p* < 0.01), and cardiogenic shock (3.8% vs. 7.2%; *p* < 0.05) between the early and the middle phases after polytrauma, respectively (Figure 2).

### 3.4. Multifactorial Shock

Multifactorial shock accounted for 9.7% (76/785) of the entire shock population in the early phase. Furthermore, 15.2% (51/335) patients diagnosed with shock were classified as having multifactorial shock in the middle phase after polytrauma (Table 3). The incidence of multifactorial shock was significantly increased in the middle phase compared to in the early phase after polytrauma (15.2% vs. 9.7%; *p* < 0.01).

#### 3.4.1. Combinations of Multifactorial Shock

**Figure 3:**
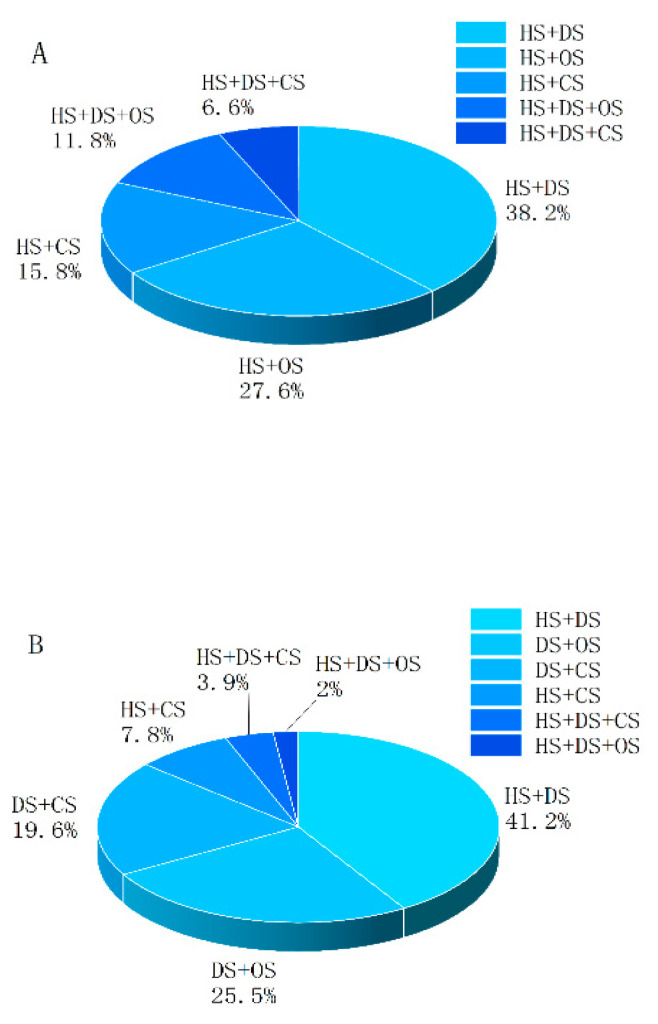
Combinations and relative frequencies of multifactorial shock in polytrauma patients in (**A**) the early phase and (**B**) the middle phase. HS: hypovolemic shock; DS: distributive shock; CS: cardiogenic shock; OS: obstructive shock.

In the present study, patients with more than one type of shock were grouped as “the multifactorial shock group” (the M-F group) and patients with only one type of shock were grouped as “the single-factor shock group” (the S-F group). The combinations of multifactorial shock are shown in Table 4. There were five combinations of multifactorial shock in the early phase after polytrauma. The combination of hypovolemic shock (HS) and distributive shock (DS) was the most common (38.2%), followed by the combination of HS and obstructive shock (OS) (27.6%). The combination of HS and cardiogenic shock (CS) accounted for 15.8%; that of HS, DS, and OS accounted for 11.8%; and that of HS, DS, and CS accounted for 6.6% (Figure 3). In the middle phase after polytrauma, six different combinations of multifactorial shock were present. The combination of HS and DS was the most common (41.2%), followed by the combination of DS and OS (25.5%). The combination of DS and CS accounted for 19.6%; that of HS and CS accounted for 7.8%; that of HS, DS, and CS accounted for 3.9%; and that of HS, DS, and OS accounted for 2.0% (Figure 3).

#### 3.4.2. Severity of Multifactorial Shock

In the early phase after polytrauma, the incidence of severe shock was significantly higher in the M-F shock group than in the S-F shock group (61.8% vs. 36.0%; *p* < 0.01) according to the MSI. Similar results were observed in the middle phase after polytrauma (76.5% vs. 40.7%; *p* < 0.01) (Table 5). 

#### 3.4.3. Complication of Multifactorial Shock

Associated complications in shock patients included acute respiratory distress syndrome (ARDS), venous thrombotic events (VTE), acute gastrointestinal injury (AGI), acute kidney injury (AKI), hospital acquired infections (HAI), and multiple organ failure (MOF). The incidence of complications in the M-F shock group was higher than that in the S-F shock group (*p* < 0.05). In the middle phase after polytrauma, patients in the M-F shock group seemed to be more likely to develop VTE (35.3% vs. 21.1%; *p* < 0.01), and 72.6% of patients in the M-F shock group developed AGI. More than four of five patients in the M-F shock group in the middle phase suffered from HAI, and 66.7% of patients showed the MOF complication (Table 5).

#### 3.4.4. Utilization of Medical Resources of Multifactorial Shock

More patients in the M-F shock group required vasoactive agent therapy and mechanical ventilation in both phases than those in the S-F shock group (*p* < 0.05). In the early phase, patients in the M-F shock group (14.8 days) were mechanically ventilated more than two-fold as often as patients in the S-F shock group (6.2 days vs. the M-F shock group; *p* < 0.01). In the middle phase after polytrauma, 98.0% of patients in the M-F shock group required mechanical ventilation and 100% of patients received vasoactive agents. Prolonged mechanical ventilation resulted in a longer length of ICU stay. The mean length of ICU stay in the M-F shock group was 16.5 days in the early phase and 19.8 days in the middle phase (Table 5).

#### 3.4.5. Mortality of Multifactorial Shock

Patients in the M-F shock group had a higher rate of mortality. In the early phase after polytrauma, the mortality of patients in the M-F shock group was 18.4% (14/76), which was significantly higher (7.7%, 54/698) than that in the S-F shock group (*p* < 0.01). Similar results were observed in the middle phase after polytrauma (13.7% [7/51] in the M-F shock group vs. 4.6% [13/280] in the S-F shock group) (Table 5).

### 3.5. Undifferentiated Shock

Some shock patients were not classified into any of the mentioned four types and were hence described as “undifferentiated shock” with the consensus of at least two of the three attending staff members. Only 11 patients suffered from undifferentiated shock in the early phase, while four patients met the definition of undifferentiated shock in the middle phase. 

## 4. Discussion

Shock is a common complication affecting approximately 63–80% of polytrauma patients [6,7]. If shock continues unchecked, it quickly results in death. According to previous reports, shock is reported as the first leading cause of death in polytrauma patients [42]. Furthermore, those who survive the initial shock insult have poor functional outcomes and significantly increased long-term mortality [43,44,45,46,47]. Further research in this field has significant implications for the treatment of polytrauma. Few studies have reported the incidence of various types of shock in polytrauma patients. To our knowledge, this is the first multi-center trial to investigate the incidence of various types of shock in different phases after polytrauma. Importantly, we characterized the incidence of multifactorial shock and emphasized that different types of shock can occur simultaneously or sequentially in polytrauma patients.

The overall incidence of shock in our study population is in line with published results [12,35]. A single-center trial by Elbaih et al. found that the most diagnostic causes of instability in polytrauma patients by RUSH (Rapid Ultrasound in Shock) are hypovolemic shock (64%), followed by obstructive shock (14%), distributive shock (12%), and cardiogenic shock (10%) [48]. Our findings are partly consistent with these results. Hypovolemic shock (83.2%) has the highest frequency in the early phase after polytrauma, followed by distributive shock (14.4%), obstructive shock (8.7%), and cardiogenic shock (3.8%). The relatively small sample size (100 cases, single-center trial) and differences in experimental design may contribute to the divergence of results; e.g., Elbaih et al. excluded patients with other types of shock and used different definitions of polytrauma. To date, there are no studies investigating the incidence of various types of shock in different phases after polytrauma.

Our study revealed that the incidences of each type of shock in trauma patients dramatically change over time. The predominant shock type in the early phase after polytrauma is hypovolemic shock due to massive bleeding trauma. Because of the widespread application of damage control surgery (DCS), most of the hemorrhage can be controlled in a timely manner [49,50]. This may partly explain why the incidence of hypovolemic shock decreased to 27.2% in the middle phase. Previous reports stated that patients who survive the initial trauma are more susceptible to infection because of immune dysregulation [51,52]. In our study, distributive shock is the main shock type in the middle phase after polytrauma and 65.4% of shock patients are septic shock cases. 

Multifactorial shock is usually considered as a rare situation in polytrauma patients, and only reported sporadically [17,18]. In this study, we report an incidence of multifactorial shock of 9.7% in the early phase and 15.2% in the middle phase after polytrauma. Subsequently, the characteristics of multifactorial shock in polytrauma patients were investigated. We report more combinations of multifactorial shock. Some examples include: (1) polytrauma patients may have hypovolemic shock from massive hemothorax as well as obstructive shock from pericardial tamponade; (2) polytrauma patients may have distributive shock (neurogenic shock) caused by cervical cord injury and hypovolemic shock caused by blunt abdominal trauma, such as massive splenic injury; (3) polytrauma patients may have distributive shock (septic shock) caused by respiratory infection and hypovolemic shock caused by stress-induced gastric ulceration; (4) patients with a history of coronary heart disease suffering from severe polytrauma may have cardiogenic shock caused by acute myocardial infarction and hypovolemic shock caused by severe penetrating trauma. 

Many studies have indicated that specific therapeutic measures are needed for each type of shock, but multifactorial shock confounds simple categorization schemes and makes the early recognition of the type of shock difficult [9,16]. According to our clinical practice experience, polytrauma patients with multifactorial shock are at a higher risk of death because the resuscitation for these patients is more difficult owing to the complicated and intractable conditions. 

The present study suggests that patients with multifactorial shock have a significantly higher mortality in the early and middle phases after polytrauma. Theoretically, more seriously injured patients are more likely to have multifactorial shock and poorer outcomes. Interestingly, there was no significant difference in ISS scores between the M-F shock group and the S-F shock group, indicating that ISS scores were not responsible for the increase in mortality of the M-F shock group in this study. Multiple mechanisms could explain the potential causal relationship between multifactorial shock and poor outcomes. First, multifactorial shock may be under-identified and undertreated. Although, in 2014, the ESICM guidelines recommended the determination of shock type to better target casual and supportive therapies, a common standard of diagnosis and treatment of multifactorial shock has not been established [9]. Second, specific therapeutic measures for each type of shock might be contradictory [53,54,55]. For example, volume expansion is needed in patients with hemorrhagic hypovolemic shock once bleeding is controlled. However, the careful administration of fluid boluses is recommended in patients with cardiogenic shock. When these two types of shock simultaneously occur, pragmatic end points for fluid resuscitation are more difficult to define. Third, this discrepancy may result from the increased incidence of severe shock in the M-F shock group. Severe shock is diagnosed according to the MSI. Accumulating evidence indicates that a higher MSI is associated with increased mortality in patients with trauma [56,57,58].

This study has some limitations. First, this was a retrospective study, and the limitations of our analysis include those inherent to retrospective databases, such as missing data, misclassification, and reporting bias. Second, despite all shock patients being classified according to the international guidelines, given the complex nature of shock, there are no unified diagnostic criteria for each type of shock. Third, although medical records, laboratory values, and imaging examination were complete in all undifferentiated shock cases, the etiology of shock in these patients remains unidentified. The development of diagnosis criteria and hemodynamic monitoring may better clarify the underlying mechanisms of undifferentiated shock in the future.

## 5. Conclusions

Our study characterized the incidence of various types of shock in different phases after polytrauma and further emphasized that different types of shock can occur simultaneously or sequentially in polytrauma patients. Although different types of shock can coexist at different time intervals, the management of traumatic shock should not be considered as a condition compartmentalized by the temporal interval only. Moreover, trauma specialists should pay much more attention to polytrauma patients with multifactorial shock with the aim of minimizing complications and mortality.

## Figures and Tables

**Table 1:** Demographics and characteristics of polytrauma patients.

Variable	Patients (*n* = 1051)
Male, *n* (%)	792 (75.4)
Age, mean (SD), y	50.2 ± 13.7
Mechanism, *n* (%)	
Traffic accident	512 (48.7)
Falls	471 (44.8)
Crush	48 (4.6)
Others	20 (1.9)
Head injury, *n* (%)	726 (69.1)
Limb injury, *n* (%)	658 (62.6)
Spine injury, *n* (%)	579 (55.1)
Chest injury, *n* (%)	488 (46.4)
Abdominal injury, *n* (%)	354 (33.7)
Pelvic injury, *n* (%)	311 (29.6)
ISS, mean (SD)	31.1 ± 9.8
GCS, mean (SD)	9.4 ± 3.5
Mortality, *n* (%)	97 (9.2)

GCS: Glasgow coma scale; ISS: injury severity score.

**Table 2:** Comparison of the shock and non-shock groups in different phases after polytrauma.

Variable	The Early Phase		The Middle Phase	
	Shock Group (*n* = 785)	Non-Shock Group (*n* = 266)	Shock Group (*n* = 335)	Non-Shock Group (*n* = 644)
Male, *n* (%)	596 (75.9)	196 (73.7)	255 (76.1)	483 (75.0)
Age, mean (SD), y	51.2 ± 14.2	49.5 ± 12.8	49.8 ± 12.5	50.5 ± 14.4
Mechanism, *n* (%)				
Traffic accident	378 (48.2)	134 (50.4)	157 (46.9)	315 (48.9)
Falls	357 (45.5)	114 (42.9)	154 (46.0)	292 (45.3)
Crush	38 (4.8)	10 (3.8)	18 (5.4)	26 (4.0)
Others	12 (1.5)	8 (3.0)	6 (1.8)	11 (1.7)
Head injury, *n* (%)	533 (67.9)	193 (72.6)	224 (66.9)	410 (63.7)
Limb injury, *n* (%)	489 (62.3)	169 (63.5)	215 (64.2)	381 (59.2)
Spinal cord injury, *n* (%)	247 (31.5)	78 (29.3)	110 (32.8)	185 (28.7)
Chest injury, *n* (%)	376 (47.9)	112 (42.1)	154 (46.0)	276 (42.9)
Abdominal injury, *n* (%)	271 (34.5)	83 (31.2)	117 (34.9)	212 (32.9)
Pelvic injury, *n* (%)	234 (29.8)	77 (29.0)	93 (27.8)	198 (30.7)
ISS, mean (SD)	33.7 ± 10.3 ^a^	27.5 ± 9.4	32.5 ± 10.2 ^a^	30.1 ± 9.5
GCS, mean (SD)	9.3 ± 3.4	9.6 ± 3.7	9.7 ± 3.2	10.1 ± 3.4
Severity of shock, *n* (%)				
Mild shock	149 (19.0) ^b^	-	45 (13.4)	-
Moderate shock	329 (41.9)	-	133 (39.7)	-
Severe shock	307 (39.1) ^b^	-	157 (46.9)	-
Mortality, *n* (%)	68 (8.7) ^a^	4 (1.5)	20 (6.0) ^a^	5 (0.8)

ISS: injury severity score; GCS: Glasgow coma scale. The early phase was defined as the period of ≤48 h after polytrauma and the middle phase was defined as the period between 48 h and 14 days; ^a^ *p* < 0.01 the shock group vs. the non-shock group in the early phase or the middle phase; ^b^ *p* < 0.05 the early phase vs. the middle phase.

**Table 3:** Incidence of various types of shock in different phases after polytrauma.

Type	The Early Phase (*n* = 785)	The Middle Phase (*n* = 335)	*p*
Hypovolemic shock, *n* (%)	653 (83.2)	91 (27.2)	<0.001
Distributive shock, *n* (%)	113 (14.4)	237 (70.7)	<0.001
Neurogenic shock	113 (14.4)	65 (19.4)	0.040
Septic shock	-	219 (65.4)	-
Anaphylactic shock	-	1 (0.3)	-
Cardiogenic shock, *n* (%)	30 (3.8)	24 (7.2)	0.022
Obstructive shock, *n* (%)	68 (8.7)	33 (9.9)	0.569
Multifactorial shock, *n* (%)	76 (9.7)	51 (15.2)	0.007
Undifferentiated shock, *n* (%)	11 (1.4)	4 (1.2)	0.782

**Table 4:** Combinations and relative frequencies of multifactorial shock in different phases after polytrauma.

Combinations	The Early Phase (*n* = 76)	The Middle Phase (*n* = 51)	*p*
HS + DS, *n* (%)	29 (38.2)	21 (41.2)	0.853
HS + OS, *n* (%)	21 (27.6)	-	-
HS + CS, *n* (%)	12 (15.8)	4 (7.8)	0.276
HS + DS + OS, *n* (%)	9 (11.8)	1 (2.0)	0.049
HS + DS + CS, *n* (%)	5 (6.6)	2 (3.9)	0.701
DS + OS, *n* (%)	-	13 (25.5)	-
DS + CS, *n* (%)	-	10 (19.6)	-

HS: hypovolemic shock; DS: distributive shock; CS: cardiogenic shock; OS: obstructive shock.

**Table 5:** Comparison of single-factor shock and multifactorial shock patients in different phases after polytrauma.

Variable	The Early Phase		The Middle Phase	
	Multifactorial Shock (*n* = 76)	Single-Factor Shock (*n* = 698)	Multifactorial Shock (*n* = 51)	Single-Factor Shock (*n* = 280)
Male, *n* (%)	55 (72.4)	533 (76.4)	36 (70.6)	215 (76.8)
Age, mean (SD), y	51.9 ± 14.8	50.8 ± 13.7	49.5 ± 12.9	50.1 ± 12.3
ISS, mean (SD)	34.5 ± 10.1	33.2 ± 10.5	33.6 ± 10.4	31.8 ± 10.0
GCS, mean (SD)	9.1 ± 3.2	9.4 ± 3.6	9.5 ± 3.1	10.0 ± 3.3
Severity of shock, *n* (%)				
Mild shock	5 (6.6) ^b^	144 (20.6)	5 (9.8)	40 (14.3)
Moderate shock	24 (31.6)	303 (43.4)	7 (13.7) ^b^	126 (45.0)
Severe shock	47(61.8) ^b^	251 (36.0)	39 (76.5) ^b^	114 (40.7)
Utilization of medical resources				
Days of ICU, mean (SD), d	16.5 ± 6.8 ^b^	7.4 ± 3.2	19.8 ± 7.3 ^b^	9.4 ± 5.1
Mechanical ventilation, *n* (%)	65 (85.5) ^b^	243 (34.8)	50 (98.0) ^b^	207 (73.9)
Days of ventilation, mean (SD), d	14.8 ± 6.5 ^b^	6.2 ± 2.1	15.7 ± 8.5 ^b^	8.3 ± 3.9
Vasoactive agents, *n* (%)	68 (89.5) ^b^	448 (64.2)	51(100.0) ^b^	228 (81.4)
Complication ^†^				
ARDS	33 (43.4) ^b^	145 (20.8)	14 (27.5) ^a^	43 (15.4)
VTE	5 (6.6)	28 (4.0)	18 (35.3) ^a^	59 (21.1)
AGI	41 (54.0) ^b^	264 (37.8)	37 (72.6) ^b^	147 (52.5)
AKI	35 (46.1) ^a^	237 (34.0)	29 (56.9) ^a^	115 (41.1)
HAI	0 (0)	0 (0)	45 (88.2)	221 (78.9)
MOF	18 (23.7) ^b^	84 (12.0)	34 (66.7) ^b^	43 (15.4)
Mortality, *n* (%)	14 (18.4) ^b^	54 (7.7)	7 (13.7) ^a^	13 (4.6)

ISS: injury severity score; GCS: Glasgow coma scale; ARDS: acute respiratory distress syndrome; VTE: venous thrombotic events; AGI: acute gastrointestinal injury; AKI: acute kidney injury; HAI: hospital acquired infections; MOF: multiple organ failure; ICU: intensive care unit; ^†^ complications were diagnosed in the early phase or the middle phase; ^a^ *p* < 0.05 and ^b^ *p* < 0.01 denote the multifactorial shock group vs. the single-factor group in the early phase or the middle phase.

## Data Availability

The data presented in this study are available on request from the corresponding author. The data are not publicly available due to ethical, legal and privacy issues.
